# Novelties in the pragmatic management of anaphylaxis in pediatric age

**DOI:** 10.1007/s00431-026-07147-3

**Published:** 2026-06-09

**Authors:** Gian Luigi Marseglia, Maria Angela Tosca, Michele Miraglia del Giudice, Sara Manti, Giorgio Ciprandi, Amelia Licari

**Affiliations:** 1 Department of Clinical, Surgical, Diagnostic and Pediatric Sciences, University of Pavia, Pavia, Italy; 2 Pediatric Clinic, Fondazione IRCCS Policlinico San Matteo, Pavia, Italy; 3 Allergy Center, IRCCS Istituto Giannina Gaslini, Genoa, Italy; 4 Department of Woman and Child and General and Specialized Surgery, University of Campania Luigi Vanvitelli, Naples, Italy; 5 Unit of Pediatrics, Department of Human Pathology in Adult and Developmental Age “Gaetano Barresi”, University of Messina, Messina, Italy; 6 Department of Medicine and Health Sciences, University of Molise, Campobasso, Italy

**Keywords:** Anaphylaxis, Children, Adrenaline, Intranasal adrenaline, Food allergy, Omalizumab

## Abstract

Anaphylaxis is a time-critical, potentially fatal systemic hypersensitivity reaction. This narrative review summarizes recent advances in the diagnosis and management of anaphylaxis in children and adolescents, with emphasis on new diagnostic frameworks, improved self-management strategies, intranasal adrenaline, and disease-modifying therapies. A narrative review was conducted using PubMed and MEDLINE, focusing on articles and guidelines published between January 2020 and March 2026. Search terms included “anaphylaxis”, “pediatric anaphylaxis”, “adrenaline”, “epinephrine”, “autoinjector”, “intranasal adrenaline”, “food anaphylaxis”, “omalizumab”, and “oral immunotherapy”. International guidelines, consensus documents, systematic reviews, pharmacokinetic studies, and pediatric studies were prioritized. Food remains the leading trigger in children, but drug, *Hymenoptera* venom, cofactor-dependent, and non-IgE-mediated mechanisms must be systematically considered. Adrenaline is underused in community settings despite being the only life-saving drug. Intranasal adrenaline represents the most visible delivery innovation: it may reduce needle-related barriers and simplify administration, but current evidence is largely based on pharmacokinetic/pharmacodynamic studies and limited pediatric clinical data. Omalizumab and oral immunotherapy are reshaping long-term risk reduction in food allergy but do not remove the need for emergency adrenaline.

*Conclusion*: The pragmatic management of anaphylaxis in children and adolescents entails self-management and hospital-based care. Intramuscular adrenaline is the first-line treatment when anaphylaxis is ongoing or recurrent, whereas adjunctive therapies should be considered on clinical grounds. Discharge recommendations should be individualized and include structured education, risk assessment, emergency planning and specialist follow-up. Intranasal adrenaline is a promising innovation, but its introduction requires clinical positioning, pharmacovigilance, cost-effectiveness evaluation, and continued emphasis on early treatment.

**Table:** 

**What is Known:** • *Intramuscular adrenaline is the first-line treatment for anaphylaxis and should not be delayed.* • *Food is the leading trigger in children, while drugs, venom, and cofactors become more relevant with age.* **What is New:** • *Intranasal adrenaline is a promising needle-free option, but pediatric evidence remains limited.* • *Omalizumab and oral immunotherapy may reduce risk but do not replace emergency preparedness.*

**What is Known:**

• *Intramuscular adrenaline is the first-line treatment for anaphylaxis and should not be delayed.*

• *Food is the leading trigger in children, while drugs, venom, and cofactors become more relevant with age.*

**What is New:**

• *Intranasal adrenaline is a promising needle-free option, but pediatric evidence remains limited.*

• *Omalizumab and oral immunotherapy may reduce risk but do not replace emergency preparedness.*

## Introduction

First described by Portier and Richet in 1902 [1], anaphylaxis is now recognized as one of the most time-sensitive emergencies in pediatric medicine, with population-based studies suggesting a substantial increase in incidence over recent decades [2].

Anaphylaxis is a severe systemic hypersensitivity reaction that may progress rapidly and cause death. Although it usually develops within minutes after exposure to a trigger and involves multiple organ systems, recent guidelines emphasize that isolated respiratory or laryngeal involvement after exposure to a known or highly probable allergen may also represent anaphylaxis and should be treated promptly [3–5]. The 2024 GA^2^LEN consensus report further simplified the definition and proposed a clinical support tool to improve recognition and standardize management across clinical settings [6].

In children and adolescents, anaphylaxis has specific diagnostic and management challenges. Food allergens are the leading triggers, but drugs, *Hymenoptera* venom, latex, cofactors, and non-IgE-mediated mechanisms must also be considered [3, 4, 7]. Recognition is particularly difficult in infants and preschool children, in whom irritability, sudden crying, lethargy, hypotonia, vomiting, or respiratory signs may be the first manifestations and subjective symptoms cannot be reliably verbalized [5, 6]. Despite consistent guideline recommendations, adrenaline remains underused because of delayed recognition, fear of adverse effects, needle phobia, poor device carriage, uncertainty about when to treat, and limited access [8–13].

Intranasal adrenaline has recently emerged as a promising needle-free option for community self-management, but its role must be interpreted cautiously because pediatric clinical data, real-world effectiveness, cost-effectiveness, and availability remain limited [13, 14].

For this narrative review, PubMed and MEDLINE were searched for articles and guidelines published between January 2020 and March 2026, prioritizing international guidelines, consensus documents, systematic reviews, pharmacokinetic and pharmacodynamic studies, and pediatric clinical evidence. This review focuses on recent diagnostic frameworks, the persistent gap between guideline recommendations and real-world adrenaline use, intranasal adrenaline, and new long-term risk-reduction strategies, including oral immunotherapy and biologic therapies.

## Pathogenetic mechanisms

Mast cells and basophils are the central effector cells in anaphylaxis and can be activated through both immunological and non-immunological pathways. The classical IgE-mediated pathway involves sensitization, binding of allergen-specific IgE to FcεRI receptors, and rapid degranulation after allergen re-exposure [15]. This mechanism is central to many food-, *Hymenoptera* venom-, latex-, and drug-induced reactions. However, IgE-mediated allergy is only one pathway. Complement activation, immune-complex mechanisms, and direct mast-cell activation through Mas-related G-protein-coupled receptor X2 (MRGPRX2) may explain reactions to blood products, biologics, opioids, vancomycin, fluoroquinolones, neuromuscular blocking agents, and radiocontrast media [15, 16].

Anaphylaxis results from the rapid release of preformed mediators, including histamine, tryptase, heparin, and chymase, followed by newly synthesized mediators such as leukotrienes, prostaglandin D2, platelet-activating factor, and pro-inflammatory cytokines. These mediators drive vasodilatation, vascular leakage, mucosal edema, bronchospasm, gastrointestinal symptoms, and cardiovascular collapse. Clinical presentation ranges from mild systemic symptoms to life-threatening respiratory or cardiovascular compromise. Neuroimmune interactions and cofactors such as exercise, fever, pain, and emotional stress may further modulate reaction threshold and severity [15–17]. This mediator network also provides the biological basis for protracted and biphasic reactions, whose frequency varies widely according to definition, setting, severity, treatment timing, and observation duration [3–6, 18]. Severe initial presentation, respiratory or cardiovascular compromise, delayed adrenaline, and repeated adrenaline dosing are consistent risk factors.

## Etiology and epidemiology

The epidemiology of anaphylaxis is heterogeneous because of differences in definitions, study settings, coding systems, healthcare access, and case ascertainment. A recent systematic review estimated a global all-cause incidence of 46–112 cases per 100,000 person-years, with an annual increase of approximately 7.4% [19]. In children, the reported incidence varies widely, reflecting differences in methodology and clinical settings [2, 8, 19].

Food-induced anaphylaxis is the leading cause of anaphylaxis in childhood, with an age-dependent allergen profile. Cow’s milk and hen’s egg predominate in infants and preschool children, whereas peanut, tree nuts, sesame, fish, shellfish, and wheat become increasingly relevant in older children and adolescents [8, 19]. Geographic variation reflects diet, food processing, migration, and environmental exposures. Emerging or increasingly recognized triggers include non-priority legumes, seeds, edible insects, plant-based meat alternatives, buckwheat, lipid transfer protein-related foods, and alpha-gal syndrome [19, 20]. Alpha-gal syndrome typically causes delayed anaphylaxis 2–8 h after mammalian meat ingestion and may be missed without targeted history-taking [20].

Beyond food, drugs and *Hymenoptera* venom become increasingly important with age. Beta-lactam antibiotics and non-steroidal anti-inflammatory drugs are common drug triggers, while radiocontrast media, neuromuscular blocking agents, perioperative agents, and biologics account for a growing proportion of iatrogenic reactions [8, 19]. Non-IgE-mediated and cofactor-dependent reactions should also be considered. Exercise, infection, fever, NSAIDs, alcohol, emotional stress, sleep deprivation, and menstrual cycle may lower the reaction threshold and convert a previously tolerated exposure into anaphylaxis [8, 19].

Adolescents and young adults are at an increased risk of severe and fatal food-induced anaphylaxis because of delayed recognition, eating outside the home, reduced supervision, reluctance to disclose allergy, poor device carriage, and delayed adrenaline use [21]. Mortality is uncommon but likely underestimated because of miscoding and underreporting. Current data mainly reflect high-income countries with established surveillance systems, whereas low-resource regions face limited diagnostic capacity, poor access to adrenaline, and lack of national registries [19, 21].

## Diagnosis and clinical features

The diagnosis of anaphylaxis remains clinical. No single symptom, biomarker, or diagnostic test can confirm or exclude anaphylaxis in the acute setting; management depends on the temporal relationship with a trigger, the pattern of organ involvement, and the likelihood of a systemic hypersensitivity reaction requiring immediate treatment [3–6].

Overall, recent international guidance is converging toward a more pragmatic diagnostic approach. WAO, EAACI, the 2023 Practice Parameter Update, and the 2024 GA^2^LEN consensus agree that anaphylaxis remains a clinical diagnosis and that treatment should not be delayed while waiting for multisystem involvement or laboratory confirmation. A relevant difference is that the most recent GA^2^LEN framework further simplifies the definition and provides a clinical support tool intended to improve recognition across different care settings [3–6, 22–25]. A major practical challenge remains the heterogeneity of severity grading systems. Several classifications have been proposed, but they may assign different severity levels to the same reaction and complicate comparisons across studies and clinical settings. In a recent analysis of positive oral food challenges, complete concordance among five severity classifications was observed only in a minority of cases, supporting the need for unified and clinically intuitive diagnostic criteria [26]. For this reason, anaphylaxis recognition should remain primarily clinical, and simple frameworks such as the EAACI diagnostic approach and the WHO ICD-11 classification may help harmonize terminology, coding, and management across specialist and non-specialist settings [3, 26].

Clinical manifestations vary by age, trigger, exposure route, comorbidities, and severity. Cutaneous and mucosal signs such as urticaria, angioedema, flushing, and pruritus are common, but their absence does not exclude anaphylaxis, particularly in rapidly progressive respiratory or cardiovascular reactions [3–6]. Respiratory involvement includes cough, wheeze, bronchospasm, stridor, dysphonia, hypoxemia, and increased work of breathing, and is a major contributor to fatal food-induced anaphylaxis, especially in patients with asthma [3–6]. Cardiovascular involvement may include hypotension, collapse, pallor, tachycardia, altered consciousness, syncope, or shock. Repetitive vomiting and abdominal pain are common in pediatric food-induced reactions [3–6, 25].

Recognition is particularly challenging in infants and preschool children, who cannot reliably report throat tightness, dizziness, pruritus, or nausea. Early signs may include sudden crying, irritability, drowsiness, hypotonia, repetitive lip licking, feeding refusal, vomiting, or reflux-like symptoms [6, 25]. The differential diagnosis includes acute urticaria or angioedema without systemic involvement, asthma exacerbation, foreign-body aspiration, vasovagal syncope, seizures, sepsis, hereditary angioedema, and mast-cell activation disorders [23]. When bronchospasm or airway compromise follows allergen exposure, adrenaline should not be delayed while alternative diagnoses are considered [3–6].

Biphasic anaphylaxis is the recurrence of symptoms after complete resolution without re-exposure. Incidence ranges from < 1% to 20%, depending on definition, setting, severity, treatment timing, and observation duration [18]. Risk factors include severe initial presentation, hypotension or hypoxemia, delayed adrenaline, repeated adrenaline doses, and protracted symptoms [5, 18]. The observation time should therefore be risk-stratified rather than uniform for all patients.

Serum tryptase can support the diagnosis, particularly in severe, atypical, perioperative, venom-, drug-induced, or medicolegal cases, but normal values do not exclude anaphylaxis, especially in children and food-induced reactions [3–6]. Acute tryptase usually peaks 30–120 min after symptom onset, may remain elevated for approximately 2 h, and generally returns to baseline within 24 h. Therefore, an acute sample should ideally be obtained within 1–2 h, with a baseline sample collected at least 24 h after symptom resolution [4, 17]. Interpretation should rely on comparison with the individual baseline value rather than on a single absolute threshold. The most widely used criterion is acute tryptase ≥ 1.2 × baseline + 2 µg/L, particularly in perioperative and mast-cell activation settings [17]. However, other validated approaches have been proposed, including an absolute increase of ≥ 2 µg/L, a relative increase to ≥ 135% of baseline in Hymenoptera venom reactions, and, more recently, a pediatric ratio-based approach in which an acute/baseline tryptase ratio > 1.74 improved diagnostic performance in children with suspected anaphylaxis [27–29]. These criteria should be interpreted in light of timing, trigger, clinical phenotype, and baseline tryptase, and should not delay acute management.

Allergy testing after clinical stabilization should be guided by clinical history. Skin-prick testing, serum specific IgE, component-resolved diagnostics, and, where available, basophil activation testing can help identify triggers and stratify risk, but negative tests do not exclude anaphylaxis when the history is compelling [3–6].

## Treatment

### Self-management

The cornerstone of anaphylaxis self-management remains early adrenaline administration. Every child or adolescent with a history of anaphylaxis, or with a clinically significant risk of future anaphylaxis, should receive an individualized emergency plan and immediate access to auto-injecting adrenaline. Intramuscular adrenaline into the mid-anterolateral thigh is the established first-line treatment, with no absolute contraindications in anaphylaxis (Table 1) [3–6]. The recommended dose depends on body weight. Available autoinjectors generally include 0.15 mg for children weighing approximately 7.5–25 kg, 0.3 mg for those weighing at least 25–30 kg, and consideration of a 0.5 mg device in adolescents or adults with higher body weight, according to local availability and national guidance [3–6].

**Table 1:** Practical management of pediatric anaphylaxis according to setting. The table distinguishes community self-management from hospital-based care and summarizes key actions to support rapid treatment, avoid unnecessary repetition of adrenaline in clinically stable patients, and guide discharge planning. Adapted from EAACI, WAO, the 2023 Practice Parameter Update, and recent adrenaline administration reviews [3–5, 31, 37]

Setting	Step	Key message
Community/self-management	Suspect anaphylaxis	Treat promptly when respiratory, cardiovascular, or severe gastrointestinal/multisystem symptoms occur after likely allergen exposure
	Adrenaline device	Use the prescribed autoinjector or intranasal adrenaline immediately, according to the action plan
	Positioning	Keep the child supine with legs elevated; allow sitting if severe breathing difficulty is present; avoid standing/walking
	Emergency activation	Call emergency services or seek urgent medical evaluation after adrenaline use
	Second dose	Give a second dose after 5–10 min if symptoms persist, worsen, or recur, or if the first dose was not delivered correctly
Hospital setting	Rapid assessment	Assess trigger, timing, previous adrenaline doses, vital signs, and ABC status
	IM adrenaline	Give immediately if anaphylaxis is ongoing, recurrent, or respiratory/cardiovascular compromise persists
	Do not repeat automatically	If stable after appropriate self-treatment, monitor rather than automatically repeating adrenaline
	Supportive care	Give oxygen for hypoxemia/respiratory distress and IV crystalloids for hypotension/shock
	Bronchodilator	Add inhaled salbutamol for persistent bronchospasm after adrenaline
	Antihistamines	Use only for residual cutaneous symptoms after ABC problems are treated
	Corticosteroids	Not first-line; routine use to prevent biphasic reactions is not supported; consider only as adjunctive therapy in selected refractory, protracted, asthmatic, or shock presentations
	IV adrenaline	Reserve infusion for refractory anaphylaxis under expert monitoring
	Observation/discharge	Use risk-stratified observation; discharge with action plan, two adrenaline doses, device training, and allergy referral

Two adrenaline doses should be prescribed whenever possible, because symptoms may persist, progress, or recur after the first administration, or because the first dose may be ineffective due to errors in device use or manipulation (Table 1) [3–6]. It is important to recommend regular checking of device expiration dates. Despite its critical importance, adrenaline remains substantially underused in self-management and community settings. The European Pediatric Anaphylaxis Registry showed correct home administration in only a minority of cases, and real-world studies continue to demonstrate gaps in prescription, carriage, and use [8, 21]. In a recent study of patients with challenge-confirmed peanut or hazelnut allergy, overall adherence to autoinjector prescription retrieval was 63.7%, with lower adherence among adolescents than younger children [12].

Barriers to adrenaline use include delayed recognition, fear of adverse effects, needle phobia, lack of confidence in device technique, social embarrassment, and over-reliance on antihistamines [10–13]. Antihistamines may improve cutaneous symptoms but do not reverse airway obstruction, bronchospasm, hypotension, or shock, and must not be used as a first-line treatment [3–6]. Education should therefore emphasize a simple rule: when anaphylaxis is suspected, adrenaline should be administered immediately and emergency services contacted. Patient engagement, when age-appropriate, and parental involvement are essential to improve awareness of disease severity, adherence to emergency recommendations, and confidence in early adrenaline use.

### Intranasal adrenaline

Intranasal adrenaline is one of the most relevant recent innovations in anaphylaxis self-management. Its rationale is practical: a needle-free device may reduce injection anxiety, improve acceptability, and facilitate earlier administration (Table 2) [13, 14].

**Table 2:** Intranasal adrenaline in pediatric anaphylaxis: opportunities and limitations. The table summarizes the clinical positioning of intranasal adrenaline as a needle-free community self-management option, highlighting its potential advantages, current evidence gaps, practical barriers, and implementation requirements [34, 35]

Domain	Practical message	Clinical implication
Rationale	Needle-free, compact, simple to administer	May reduce injection anxiety and bystander hesitation
Evidence base	Mainly PK/PD data; pediatric clinical data still limited	Promising, but not yet supported by large real-world comparative studies
Current role	Community self-management option for eligible patients	Complementary to established emergency pathways
Not a replacement for	IM adrenaline in professional algorithms; IV adrenaline in refractory anaphylaxis	Standard emergency treatment pathways remain essential
Dosing	Single-use device; two doses still required	Families should have access to a second dose
Limitations	Limited pediatric experience; uncertain performance in selected nasal conditions	Individual assessment is needed before prescribing
Practical barriers	Availability, cost, reimbursement, pharmacy access, device familiarity	Implementation will vary across countries and healthcare systems
Potential candidates	Needle phobia, previous AAI non-use, adolescents reluctant to carry injectables, need for bystander administration	May improve acceptability and early treatment
Education	Action plan and device training remain mandatory	Device choice does not replace standard anaphylaxis education

The first approved intranasal formulation, neffy®/EURneffy®, uses absorption-enhancing technology to achieve clinically relevant systemic exposure after nasal administration. Other formulations, including liquid and powder products, remain under development [13, 14]. Pharmacokinetic and pharmacodynamic studies suggest that intranasal adrenaline can achieve systemic exposure comparable to injectable devices, and early pediatric data are encouraging. A small phase 3 study in 15 children who developed anaphylaxis during oral food challenges reported symptom resolution with a median time of 16 min and no serious adverse events [14]. However, these data were obtained in controlled medical settings and cannot fully reproduce community anaphylaxis, where delayed recognition, panic, asthma, and variable caregiver performance may influence outcomes. Intranasal adrenaline should therefore be framed as a promising option for community self-management in eligible patients, not as a replacement for intramuscular adrenaline in professional emergency algorithms or for intravenous adrenaline infusion in refractory anaphylaxis [3–6, 13, 14].

**Fig. 1:**
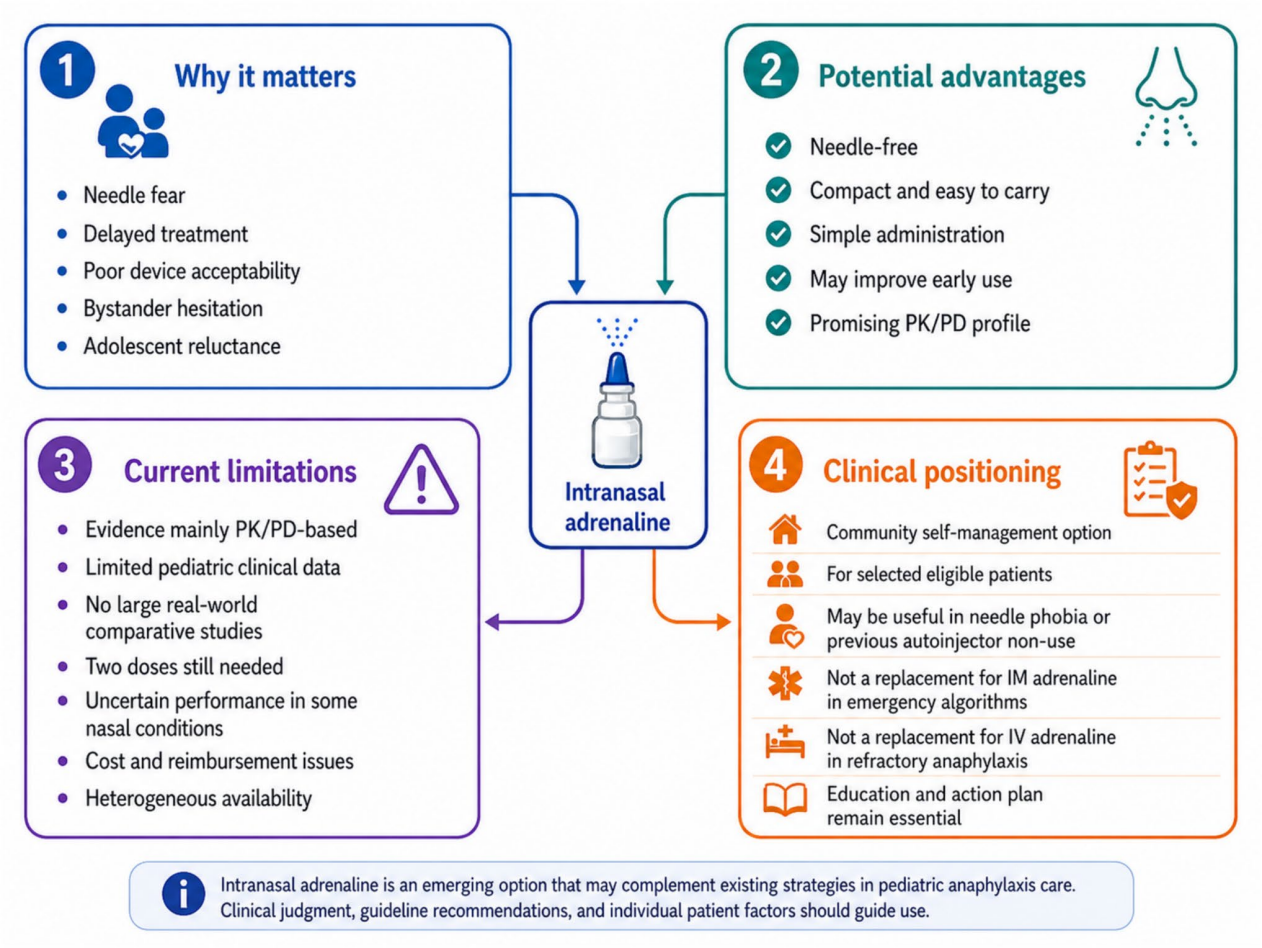
Intranasal adrenaline in pediatric self-management: promise and limits. Schematic overview of the rationale, potential advantages, current limitations, and clinical positioning of intranasal adrenaline in children and adolescents at risk of anaphylaxis [13, 14]. Intranasal adrenaline may reduce barriers related to needle fear and device acceptability, but current evidence remains mainly pharmacokinetic/pharmacodynamic, with limited pediatric clinical and real-world comparative data. Its use should be considered complementary to established emergency pathways. Based on recent pediatric reviews and evidence [13, 14]

Current limitations include the predominance of pharmacokinetic/pharmacodynamic evidence, limited pediatric clinical experience, lack of large real-world comparative studies, single-use devices requiring access to two doses, and uncertain performance in some nasal conditions [13, 14]. Heterogeneous availability, cost, and reimbursement remain additional practical barriers [30–32]. These opportunities and limitations are summarized in Fig. 1. Regulatory approval and practical availability are rapidly evolving and differ substantially across countries; selected current regulatory and access details are provided in Table 3. Device choice should be individualized, particularly during early implementation. Intranasal adrenaline may be especially useful for patients with needle phobia, previous failure to use an autoinjector, adolescents reluctant to carry injectable medication, or settings where ease of bystander administration is critical.

**Table 3:** Current regulatory and practical availability of intranasal epinephrine. Overview of selected regulatory approvals, pediatric indications or weight ranges, and practical access considerations. Regulatory status, reimbursement, and commercial availability are time-sensitive and should be rechecked before submission and publication

Country/region	Product	Regulatory status	Pediatric indication/weight range	Practical considerations
United States	neffy®	FDA-approved	Approved for patients weighing ≥ 15 kg; 1 mg for 15 to < 30 kg and 2 mg for ≥ 30 kg	Commercially available by prescription; cost and coverage vary; two doses remain necessary
European Union/EEA	EURneffy®	EMA/European Commission authorization	Adults and children from 4 years of age weighing ≥ 15 kg	Central authorization does not ensure immediate national availability; pricing and reimbursement are country-specific
Germany	EURneffy®	First European market launch reported in 2025	Adults and children according to approved EU labeling	First confirmed EU launch; access depends on national pricing and reimbursement
United Kingdom	EURneffy®	MHRA-approved in 2025	Approved for emergency treatment of anaphylaxis; local label and age/weight details should be checked before prescribing	Approval does not automatically guarantee universal NHS availability; supply and prescribing pathways may evolve
Australia	neffy®	TGA-approved	Registered 1 mg and 2 mg doses; weight-based use reported	Available/being introduced; not necessarily reimbursed through national schemes at launch
Japan	neffy®	PMDA-approved	1 mg and 2 mg doses approved for patients > 15 kg	Commercial launch and local access depend on partner distribution and reimbursement
China	neffy®	NMPA-approved	2 mg for adults and children > 30 kg	Reported as first community-use epinephrine product in China; commercial access still evolving
Canada	neffy®	Health Canada-approved in 2026	2 mg for adults and children > 30 kg	Availability expected after approval; 1 mg pediatric dose for 15–30 kg not yet approved at time of writing
Italy	EURneffy®	Covered by EU authorization	EU indication applies once nationally marketed	National availability, AIFA pricing/reimbursement, and pharmacy access should be verified before submission/publication

The regulatory details in this table are time-sensitive: FDA labeling supports use of neffy in patients ≥ 15 kg, EMA information states that Eurneffy is indicated in adults and children from 4 years weighing ≥ 15 kg, Medicines and Healthcare products Regulatory Agency (MHRA) approved EURneffy in July 2025, and Germany was reported as the first EU launch market in June 2025. Australia, Japan, China, and Canada have separate national approvals or launch pathways that should be rechecked before journal submission

Other alternative adrenaline delivery systems are also under investigation. Needle-free injection devices have been developed to improve intramuscular delivery and potentially overcome limitations related to needle length, injection technique, and needle-related anxiety, with early studies showing promising device-performance results [33]. Sublingual adrenaline formulations have also been explored as non-invasive emergency treatment strategies, although current clinical evidence remains preliminary. At present, these approaches should be considered investigational and are not part of routine anaphylaxis management [33, 34].

### Hospital-based treatment

In the emergency department, management should begin with rapid clinical assessment, including the history of the reaction, suspected trigger, time from exposure, timing and number of adrenaline doses already administered, vital signs, and current airway, breathing, and circulation status. If anaphylaxis is ongoing, symptoms recur, or respiratory or cardiovascular compromise persists, intramuscular adrenaline should be administered without delay. Treatment should not be postponed by attempts to obtain intravenous access, administer antihistamines or corticosteroids, perform laboratory testing, or observe whether symptoms improve spontaneously (Table 1) [3–6]. Conversely, in a child who has already received one or two appropriate adrenaline doses and is clinically stable with complete or near-complete symptom resolution, immediate repeat adrenaline is not required; close monitoring and risk-stratified observation are appropriate.

Initial supportive measures include removal of the trigger when feasible, calling for help, correct positioning, airway assessment, high-flow oxygen for respiratory distress or hypoxemia, and early intravenous access in patients with cardiovascular compromise. Fluid replacement by intravenous crystalloid boluses of 20 mL/kg should be administered for hypotension or shock and repeated according to clinical response. Inhaled salbutamol may be added for persistent bronchospasm after adrenaline, but it does not treat upper-airway edema, hypotension, or systemic vascular collapse [3–6].

Antihistamines and corticosteroids must never delay adrenaline administration. H1-antihistamines have a limited role in the treatment of anaphylaxis and should not be used to manage respiratory or cardiovascular manifestations. Their use may be considered only for residual cutaneous symptoms, such as urticaria or pruritus, after life-threatening features have been treated [4]. Systemic corticosteroids do not treat the acute vasoactive phase of anaphylaxis and should not be used as first-line therapy or as a routine strategy to prevent biphasic reactions, as the current evidence does not support this indication and some studies have raised concerns regarding possible unfavorable outcomes associated with their routine use [3–6, 10, 17, 18]. However, they may be considered adjunctive therapy in selected cases, such as refractory anaphylaxis, persistent bronchospasm or asthma exacerbation, ongoing shock, or protracted symptoms.

Refractory anaphylaxis is suspected when respiratory or cardiovascular compromise persists despite appropriate intramuscular adrenaline and adequate fluid resuscitation. In this setting, intravenous adrenaline infusion should be started under continuous cardiac and blood-pressure monitoring by clinicians experienced in pediatric resuscitation [3–6]. Glucagon may be considered in patients receiving beta-blockers who respond poorly to adrenaline, although this is uncommon in children. Additional vasopressors may be required in refractory vasodilatory shock in an intensive care setting [3–6].

**Fig. 2:**
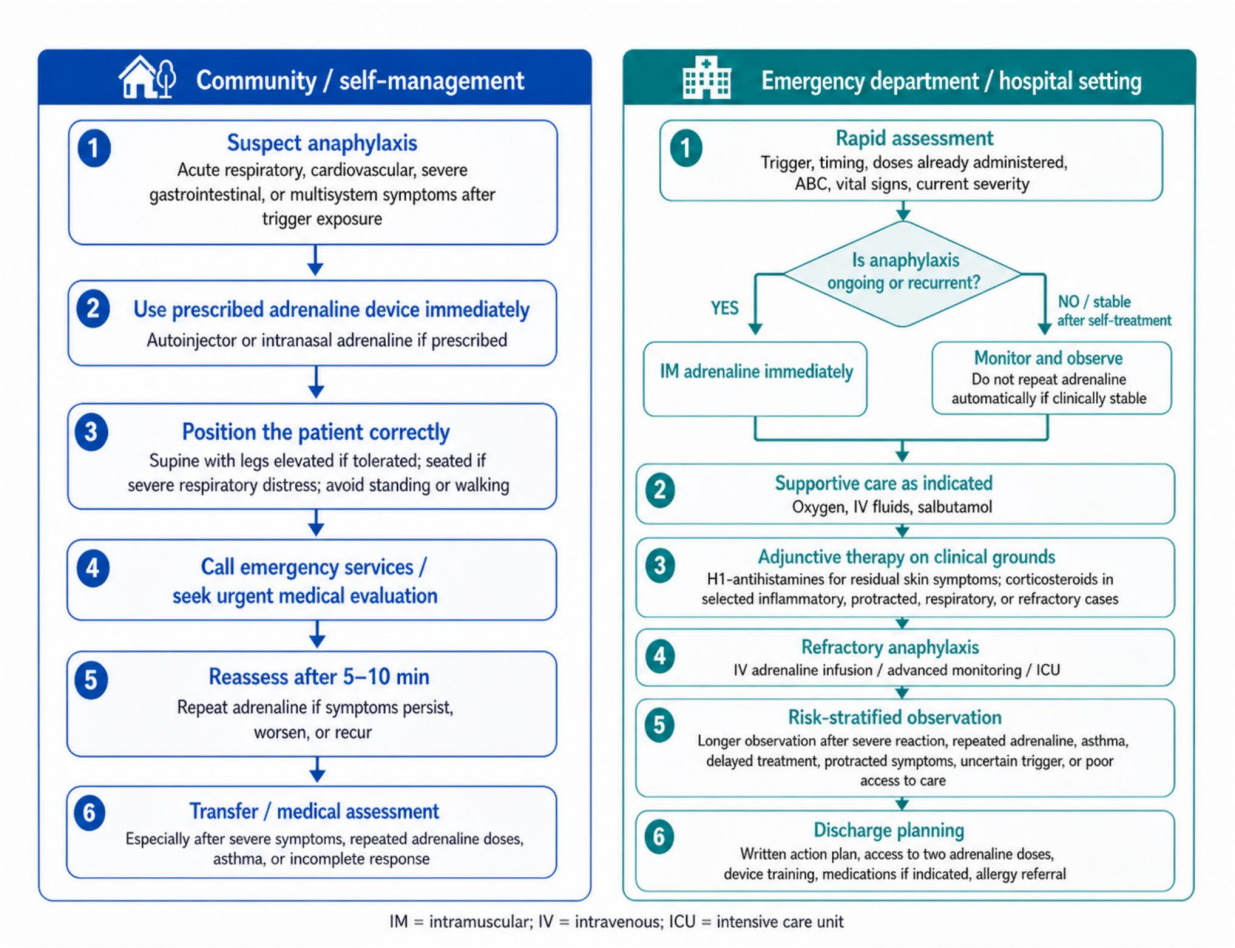
Pragmatic management of pediatric anaphylaxis according to setting. The algorithm distinguishes community self-management from emergency department/hospital care. In the community, prescribed adrenaline devices should be used immediately when anaphylaxis is suspected. In hospital, management begins with rapid assessment of clinical status, trigger, timing, and doses already administered. Intramuscular adrenaline is indicated when anaphylaxis is ongoing or recurrent, whereas clinically stable patients who have already self-treated require monitoring and risk-stratified observation rather than automatic repeat dosing. Adapted from current international recommendations [3–6]

Observation after resolution should be individualized. A shorter observation may be appropriate after a prompt response to a single adrenaline dose and complete symptom resolution. Prolonged monitoring, up to 24 h or longer, should be considered after severe respiratory or cardiovascular involvement, repeated adrenaline dosing, delayed treatment, protracted symptoms, poorly controlled asthma, uncertain trigger, remote residence, poor family reliability, or limited access to emergency care [5, 6]. A practical algorithm distinguishing community self-management from hospital-based management is shown in Fig. 2.

Discharge is a critical phase of anaphylaxis management and should be individualized according to reaction severity, response to treatment, comorbidities, distance from emergency care, family reliability, and access to specialist follow-up. Before discharge, patients and caregivers should receive clear written instructions, including trigger avoidance, warning symptoms, positioning, emergency service activation, and when to administer a second adrenaline dose. Access to two adrenaline doses should be confirmed whenever possible; if definitive prescription is not completed in the emergency department, urgent allergy referral should ensure device selection, prescription, and structured training [3–6].

Adjunctive medication should be prescribed only when clinically indicated. Non-sedating H1-antihistamines may be used for persistent cutaneous symptoms. Corticosteroids may be considered in patients with protracted symptoms, asthma exacerbation, significant bronchospasm, or refractory reactions. All patients should receive practical education with trainer devices and a written action plan, and schools or caregivers should be informed when appropriate [3–6].

### Long-term management

Long-term management includes trigger identification, allergen avoidance, cofactor recognition, risk stratification, education, psychological support, and, where available, disease-modifying therapy. Management should be individualized according to the trigger, previous reaction severity, age, asthma control, comorbidities, family preferences, and access to specialist care.

A relevant recent shift is the move from avoidance-only strategies toward individualized risk-reduction approaches. Oral immunotherapy, biologic therapy, venom immunotherapy, and improved cofactor management may increase reaction thresholds, reduce the impact of accidental exposures, and improve quality of life. However, none of these strategies eliminates the need for emergency preparedness, including written action plans and ready access to adrenaline.

For food-induced anaphylaxis, avoidance should be precise and evidence-based rather than unnecessarily restrictive. Families should be educated on label reading, cross-contact, eating outside the home, school meals, sports, travel, and cofactors such as exercise, NSAIDs, fever, infection, alcohol in adolescents, sleep deprivation, and emotional stress [35]. Oral immunotherapy consists of the regular administration of increasing doses of the culprit allergen under specialist supervision, to induce desensitization and raise the reaction threshold. It is increasingly used for selected IgE-mediated food allergies, particularly peanut, cow’s milk, and egg, but should be performed only in specialized centers [35–37]. Families should be informed that OIT reduces the risk of reactions after accidental exposure but does not replace allergen avoidance, action plans, or adrenaline availability.

Venom immunotherapy is the treatment of choice for Hymenoptera venom anaphylaxis in patients with systemic reactions and confirmed sensitization. It provides high protection rates and should usually continue for at least 3–5 years; longer treatment may be required in patients with severe reactions, mastocytosis, elevated baseline tryptase, or high-risk exposure [38].

Drug desensitization may be considered when the culprit drug is medically essential and no equivalent alternative exists, but only in specialized settings with resuscitation facilities [3].

### Biological therapy: omalizumab

Omalizumab, a monoclonal anti-IgE antibody, has introduced a new risk-reduction strategy in IgE-mediated food allergy. In February 2024, it received FDA approval to reduce allergic reactions, including anaphylaxis, after accidental exposure to one or more foods in patients aged ≥ 1 year [39]. Its role is not emergency treatment: patients must continue allergen avoidance and carry adrenaline. In the OUtMATCH trial, 66.9% of omalizumab-treated participants tolerated at least 600 mg of peanut protein, compared with 6.8% in the placebo group; protection also extended to other food allergens in many participants [39]. Omalizumab may be particularly relevant for patients with multiple food allergies, a high risk of accidental exposure, severe anxiety, poor suitability for OIT, or previous reactions despite careful avoidance. Recent pediatric evidence further supports the role of omalizumab as a pragmatic risk-reduction strategy in selected children with severe IgE-mediated food allergy. In a real-life observational study including food-allergic children with severe asthma and previous anaphylaxis, omalizumab significantly increased reaction thresholds, reduced food-related reactions, improved food allergy-related quality of life, and allowed partial or complete reintroduction of culprit foods in many patients during treatment [40]. Similarly, recent data from the OUtMATCH extension study showed that, after omalizumab treatment, many children and adolescents were able to introduce allergenic foods into their diet, although adverse reactions, including anaphylaxis and eosinophilic esophagitis, still occurred and some patients returned to avoidance strategies [41]. These findings suggest that omalizumab may increase protection against accidental exposures and facilitate dietary liberalization in selected patients, but it does not induce definitive tolerance or eliminate the need for emergency preparedness and immediate access to adrenaline. Its availability, reimbursement, indication, treatment duration, and cost-effectiveness vary by country and should be discussed within specialist allergy services [35]. Beyond clinical outcomes, basophil-based biomarkers may help characterize response to omalizumab. In asthma and chronic spontaneous urticaria, omalizumab has been shown to modulate basophil homeostasis, FcεRI expression, intracellular signaling, activation, and cytokine release, suggesting that basophils may contribute to treatment response and serve as potential pharmacodynamic biomarkers [42]. In food allergy, evidence remains limited; however, a recent study in peanut-allergic children showed that basophil activation testing and basophil histamine release assays reflected omalizumab-induced reductions in basophil reactivity and correlated with improved challenge outcomes, supporting their potential role as investigational tools to predict or monitor response [43]. Further studies are needed before these biomarkers can be incorporated into routine clinical decision-making.

## Prevention and quality of life

Prevention in pediatric anaphylaxis includes prevention of sensitization, severe outcomes, delayed treatment, unnecessary restriction, psychosocial harm, and inequitable access to care. Early introduction of allergenic foods, especially peanut and cooked egg, has reframed food allergy prevention and is now preferred over delayed introduction during complementary feeding when developmentally appropriate [35, 44, 45]. However, early introduction does not prevent all food-induced anaphylaxis and does not address emerging or region-specific allergens; surveillance, accurate diagnosis, and access to specialist care remain essential [7, 35].

Education is a core component of prevention. Families should receive written action plans and repeated practical training on symptom recognition, adrenaline use, positioning, emergency activation, and second-dose indications [3, 5, 10]. Schools require accessible action plans, trained staff, rapidly available devices, and clear responsibility for treatment. Policies should balance safety with inclusion: broad food bans may create false reassurance, whereas individualized risk assessment, supervised eating for younger children, allergen-aware meal planning, and anti-bullying measures are more sustainable [3, 7, 8]. Psychological support may reduce maladaptive avoidance, improve treatment confidence, and support age-appropriate autonomy.

Food allergy and anaphylaxis have a substantial impact on quality of life. Parents often report vigilance, anxiety, and difficulty delegating care; children and adolescents may experience social exclusion, bullying, dietary restriction, and fear of eating outside the home [46]. Psychological support should be integrated when anxiety impairs nutrition, school participation, independence, or adherence. Precautionary allergen labeling remains a major unresolved problem because voluntary statements are inconsistently applied and often do not reflect quantified risk. Risk-based systems such as the Voluntary Incidental Trace Allergen Labelling (VITAL®) framework may reduce unnecessary avoidance while maintaining safety, but wider harmonization is needed [47].

## Conclusions

Pediatric anaphylaxis management is evolving through broader diagnostic frameworks, improved self-management, new adrenaline delivery systems, and disease-modifying strategies, but its central principle remains unchanged: suspected anaphylaxis must be recognized early and treated promptly with intramuscular adrenaline. Improved diagnostic frameworks, better understanding of IgE- and non-IgE-mediated mechanisms, and greater awareness of cofactors may help clinicians identify reactions that might otherwise be missed, especially in infants, young children, and adolescents. The main gap in care remains delayed or absent adrenaline use in real-life settings. Antihistamines and corticosteroids must not delay first-line treatment. Corticosteroids may be considered adjunctive therapy on clinical grounds, particularly in refractory reactions, persistent bronchospasm or asthma exacerbation, ongoing shock, or protracted symptoms [3–6]. Intranasal adrenaline is a promising innovation for community self-management, but the evidence remains mainly pharmacokinetic/pharmacodynamic, and real-world effectiveness, cost, reimbursement, and availability require further evaluation [13, 14]. Self-management and community management require adequate education and engagement. Long-term strategies, including oral immunotherapy, omalizumab, and venom immunotherapy, can reduce the risk but do not eliminate the need for allergen awareness, emergency planning, and ready access to adrenaline [32, 33]. The goal of care is to prevent fatal outcomes while enabling children and adolescents to participate safely and confidently in daily life.

## Data Availability

No datasets were generated or analysed during the current study.

## Abbreviations

FDA: Food and Drug Administration
FcεRI: High-affinity immunoglobulin E receptor
GA^2^LEN: Global Allergy and Asthma Excellence Network
IgE: Immunoglobulin E
IM: Intramuscular
IV: Intravenous
MRGPRX2: Mas-related G-protein-coupled receptor X2
NSAIDs: Non-steroidal anti-inflammatory drugs
OIT: Oral immunotherapy
VITAL: Voluntary Incidental Trace Allergen Labelling
WAO: World Allergy Organization
